# The potential long-term impact of the COVID-19 outbreak on patients with non-communicable diseases in Europe: consequences for healthy ageing

**DOI:** 10.1007/s40520-020-01601-4

**Published:** 2020-05-26

**Authors:** Katie Palmer, Alessandro Monaco, Miia Kivipelto, Graziano Onder, Stefania Maggi, Jean-Pierre Michel, Rita Prieto, Georgia Sykara, Shaantanu Donde

**Affiliations:** 1 Oliba, Rome, Italy; 2 Catholic University of the Sacred Heart, Rome, Italy; 3 HEC, 1, Rue de la Liberation Jouy en Josas, Paris, France; 4 Division of Clinical Geriatrics, Department of NVS, Center for Alzheimer Research, Karolinska Institutet, Karolinska University Hospital, Theme Aging, Stockholm, Sweden; 5 Institute of Public Health and Clinical Nutrition, University of Eastern Finland, Kuopio, Finland; 6 Ageing and Epidemiology (AGE) Research Unit, School of Public Health, Imperial College London, London, UK; 7 Department of Cardiovascular, Endocrine-Metabolic Diseases and Aging, Istituto Superiore di Sanità, Rome, Italy; 8 CNR-Neuroscience Institute, Aging Branch, Padua, Italy; 9 Department of Geriatrics and Rehabilitation, Medical University of Geneva, Geneva, Switzerland; 10 Pfizer GEP SLU, Upjohn, Madrid, Spain; 11 Medical Affairs, Upjohn Hellas Ltd (Division of Pfizer), Athens, Greece; 12 Upjohn (Division of Pfizer), Surrey, UK

**Keywords:** COVID-19, SARS-coV-2, Chronic diseases, NCD, Elderly, Frailty, Coronavirus

## Abstract

The early stages of the COVID-19 pandemic have focused on containing SARS-CoV-2 infection and identifying treatment strategies. While controlling this communicable disease is of utmost importance, the long-term effect on individuals with non-communicable diseases (NCD) is significant. Although certain NCDs appear to increase the severity of COVID-19 and mortality risk, SARS-CoV-2 infection in survivors with NCDs may also affect the progression of their pre-existing clinical conditions. Infection containment measures will have substantial short- and long-term consequences; social distancing and quarantine restrictions will reduce physical activity and increase other unhealthy lifestyles, thus increasing NCD risk factors and worsening clinical symptoms. Vitamin D levels might decrease and there might be a rise in mental health disorders. Many countries have made changes to routine management of NCD patients, e.g., cancelling non-urgent outpatient visits, which will have important implications for NCD management, diagnosis of new-onset NCDs, medication adherence, and NCD progression. We may have opportunities to learn from this unprecedented crisis on how to leverage healthcare technologies and improve procedures to optimize healthcare service provision. This article discusses how the COVID-19 outbreak and related infection control measures could hit the most frail individuals, worsening the condition of NCD patients, while further jeopardizing the sustainability of the healthcare systems. We suggest ways to define an integrated strategy that could involve both public institutional entities and the private sector to safeguard frail individuals and mitigate the impact of the outbreak.

## The COVID-19 pandemic

In December 2019, the first confirmed case of SARS-CoV-2 infection was reported in Wuhan China [1], leading to an outbreak that was declared a pandemic by the World Health Organization (WHO). Although we are currently in the early stages of the outbreak in Europe, the disease has already caused thousands of deaths and led to Government-enforced quarantine measures in several countries. Although the current focus of Governments and health ministries is to employ urgent measures to slow SARS-CoV-2 infection rates and minimize the number of infected individuals, the pandemic will potentially also have a substantial long-term impact on people with non-communicable diseases (NCDs) in Europe. While the focus on controlling this communicable disease is of utmost importance, the long-term effect on NCDs is also of vital significance. It could further jeopardize the sustainability of healthcare systems by worsening the condition of patients with chronic conditions, as discussed below. The pandemic could potentially affect Quality Adjusted Life Years making the socio, clinical, and economic impact of NCDs even worse. There are almost 94 million persons aged 65 and over in the EU, accounting for an 18.5% share of the population [2], and the majority of persons in this age group have multimorbidity, defined as multiple concurrent NCDs [3]. Here, we briefly discuss the various ways in which the pandemic may impact NCD management and affect patient outcomes in Europe.

## Change in NCD prevalence due to mortality in persons infected with SARS-CoV-2

Initial reports indicate that mortality in persons infected with SARS-CoV-2 appears to be higher in men and persons with NCDs; a meta-analysis of the first cases early in the outbreak suggest a case-fatality rate of approximately 7% [4], although this estimated rate is likely to reduce when more precise calculations of infected individuals that include asymptomatic or minimally symptomatic cases become available [5]. Moreover, it is still unclear how many nursing home residents have died from causes related to SARS-CoV-2 infection or the number of deaths from people who avoided seeking inpatient medical care for fear of getting infected. Many deaths involve men and individuals with preexisting medical conditions such as coronary diseases, hypertension, diabetes and active cancer, often with multimorbidity [6, 7]. The first reports mainly come from China and Italy [6, 7] but if these mortality patterns continue in other countries throughout the outbreak this has the potential to have a long-term effect on the prevalence of some NCD prevalence rates; NCDs that are associated with mortality in SARS-CoV-2 infected patients, such as cardiovascular disorders and diabetes may subsequently decrease in prevalence at the population level. Although there is no peer-reviewed scientific evidence on this issue yet, initial reports from scientific societies in Italy [8, 9] and Spain [10] suggest a substantial reduction in admission for stroke or Myocardial infarction since the start of the outbreak, for example. In Spain, cardiologists have seen a 40% reduction in heart attack treatments and a decrease in the number of diagnostic procedures [10]. It is imperative to establish the reasons behind these patterns. One hypothesis is that individuals do not go to the hospital even if they need to or that such cases are admitted to the hospital as a SARS-CoV-2 admission. There is also a shortage of healthcare staff to cover both SARS-CoV-2 related illness and all other routine medical care, as discussed below.

## The role of SARS-CoV-2 infection on NCD progression

In patients with NCDs, a concurrent SARS-CoV-2 infection may affect the progression and health outcome of their pre-existing chronic disorder. Evidence from other respiratory infections such as seasonal influenzas, for example, show that the infection can exacerbate chronic NCD conditions (e.g., asthma, chronic obstructive pulmonary disease, congestive cardiac failure) [11] and are associated with functional decline in older persons [12]. Conditions such as community-acquired pneumonia are associated with cognitive decline [13]. Moreover, functional deterioration occurs quickly after hospitalization in a significant proportion of older patients after an acute event such as pneumonia and typically fails to improve by the time of discharge [14]. This evidence highlights the need to monitor NCD progression in those infected with SARS-CoV-2 to examine how it affects NCD symptoms as well as short-and long-term functional and cognitive decline.

## The long-term impact of infection control measures on older persons with NCDs

During the first months of 2020, governments in many European countries initiated containment measures with different approaches ranging from restricting population movement and increasing social distancing, to implementing voluntary or enforced isolation and quarantine measures [15]. Self-isolation recommendations were particularly targeted to older persons and/or to individuals with chronic medical conditions, who have a higher risk of negative health outcomes resulting from SARS-CoV-2 infection. However, these measures have the potential to impact short- and long-term NCD management and progression from various perspectives, as discussed below.

### Reduced physical activity levels and other lifestyle factors

One consequence of quarantine measures will be a decrease in physical activity levels in many individuals. The closure of gyms, swimming pools, and exercise clubs in addition to laws limiting access to outdoor space and free movement will inevitably reduce opportunities to exercise. This is of particular importance to NCD patients, where physical activity is essential for controlling symptoms and risk factors such as obesity, hypertension, and elevated glucose levels [16]. Exercise is also important for reducing sarcopenia, falls, and fall-related injuries [17, 18]. Further, cardiorespiratory fitness is also associated with cognitive functioning in older persons [19]. In situations where opportunities for aerobic exercise training are limited, alternatives such as resistance exercise training may need to be proposed as potential alternatives, as evidence suggest that it may be as effective for reducing the risk of several chronic diseases [9]. Access to fresh food may be limited leading to changes in diet, which can affect a number of health outcomes including cognition [20]. Nutrition, exercise, cognitive training, and management of metabolic and vascular risk factors are all important for maintaining cognitive functioning and reducing the risk of chronic diseases in older persons [21].

### Reduced social contact and its effect on loneliness and mental health disorders

Quarantine measures will lead to reduced social contact and increased loneliness in older individuals. This may lead to a rise in mental health disorders, such as depression, and affect outcomes of patients who have existing mental health conditions. According to the EU statistics on income and living conditions, 13.4% of households in the EU in 2013 were composed of a single person aged 65 or over. Physical and social environments are related to loneliness and mental health factors in persons aged over 50, with social cohesion and social participation playing an important role [22]. Reduced social network, isolation, and loneliness can lead to generalized anxiety and major depression disorders in older persons [23] and have been shown to negatively affect a range of health outcomes [24], including increased healthcare utilization and mortality as well as malnutrition and vitamin D deficiency [25], which have important implications as discussed below. Further, people with existing mental health disorders may suffer during quarantine periods due to difficulties accessing regular outpatient visits for evaluations and prescriptions and they might be more influenced by the emotional responses related to the pandemic that could lead to relapse or worsening of mental health symptoms [26].

### Reduction in vitamin D

Home-isolation is also likely to lead to a reduced number of hours spent outdoors, which may affect vitamin D levels. Low sunlight exposure periods are associated with vitamin D concentrations [27]. This can have relevant health consequences as low levels of vitamin D are associated with numerous NCDs [28] and a higher prevalence of multimorbidity [29]. Further, evidence exists on a link between vitamin D deficiency and impaired immune function, potentially leading to autoimmunity and increased risk of infections [30].

### Changes to routine management of NCD patients

During the SARS-CoV-2 outbreak, healthcare systems began postponing and scaling down some aspects of routine NCD management, outpatient visits, and non-urgent surgery to avoid unnecessary hospital visits, reduce the burden on hospitals, and decrease infection risk [31]. Although there are no data available yet on this issue, it is likely that many NCD patients have decreased access to outpatient visits and one-on-one clinical advice, and, in some cases, there may be a shortage of medicines. Further, some patients may be reluctant to seek care due to fears of infection in healthcare settings. The situation is further exacerbated by the preexisting European shortage of skilled healthcare workers [32] and that many healthcare workers have been infected with SARS-CoV-2, which affects staffing levels. In the short-term this has important consequences for integrated care programs, which are a vital element of the care of persons with NCDs, particularly patients with multimorbidity [33, 34]. Lack of integrated support from local health units and healthcare systems, whose focus in now on managing the COVID-19 crisis, may undermine welfare, particularly hitting vulnerable groups such as the elderly and persons with frailty and NCDs. In the long-term, reduced NCD management may have devastating consequences to some individuals, particularly those with multiple or more severe conditions who require regular symptom monitoring and adjustment of complex drug regimens. In addition, the redistribution of healthcare resources to fight the SARS-CoV-2 outbreak is likely to have long term financial effects on national health systems throughout Europe, affecting patients both with non-communicable and communicable diseases. Indeed, it is also important that vaccination for other communicable diseases are not neglected as they are also of crucial importance for fighting against emerging and re-emerging infectious diseases and for maintaining better health and functioning in older individuals.

### Medication adherence

Integrated care and clinical monitoring of NCD patients are essential for maintaining medication adherence. The scaling down of outpatient visits, as mentioned above, in addition to quarantine measures that may affect access to pharmacies may have relevant clinical implications in terms of drug adherence. Further, as rapid research into the clinical management of SARS-CoV-2 infected individuals continues and the media report premature findings to the general public there may be dangerous consequences to patients who might start to self-manage their NCD medications. For example, in March 2020 reports emerged on potential adverse effects of angiotensin-converting enzyme inhibitors (ACE-i) or Angiotensin Receptor Blockers (ARB) on the risk of infection and the severity of SARS-CoV2, leading to the publication of a position statement from the European Society of Cardiology [35] urging physicians and patients not to discontinue antihypertensive treatment with ACEi or ARBs. Adherence to medication regimens and continuity of treatment is essential for the management of NCDs in older persons to avoid long-term negative health outcomes [36, 37], and pharmacists may need to play a role in this, hopefully with the support of computerized prescription registries.

### Effects on NCD research

Many research projects on NCDs in Europe have been either halted or postponed during the COVID-19 outbreak to drain strategic resources (structural, financial, and human) from the NCD epidemic and redirect them to the COVID-19 pandemic. In addition, several **c**linical studies have been paused because healthcare systems are under pressure and clinical staff do not have time to complete research protocols. Many laboratory-based projects have been temporarily suspended due to infection risks to staff, with numerous universities also closing across Europe. The postponement of ongoing NCD research projects that focus on identifying risk factors and treatment options for NCDs will likely derail progress in this area by further delaying cost-effective interventions.

## Future strategies to reduce the impact of the COVID-19 pandemic on persons with NCDs

We are at the start of this unprecedented situation, and currently we can only speculate on the potential long-term impact that the COVID-19 outbreak will have on individuals with NCDs. In the meantime, we should urgently address the basic needs of NCD patients by identifying priority actions to support them in the management of their chronic conditions, both during the existing emergency and in the medium/long term. We need to define an integrated strategy that could involve both private companies and institutional entities to safeguard frail individuals and mitigate the impact of the outbreak. Politicians at national and European levels also need to prioritize this within their planning strategies during and after the pandemic. Actions to minimize the potentially devastating impact of the COVID-19 outbreak on older persons with NCDs should leverage on the use of cost-effective tools (e.g., digital health technologies) that could ensure access to both critical information (such as access to healthcare resources, clinical and lifestyle guidance, the importance of continuing vaccination programmes for other communicable diseases) and monitoring from healthcare professionals (including telemedicine). A common effort is needed to ensure the continuity of healthcare services, where the outbreak emergency could coercively push the adoption of sustainable solutions toward the decentralization of social and clinical assistance. These actions may include: awareness and education campaigns; tools to facilitate communication with healthcare professionals; improved access to institutional health communication and; better access to local and social support activities. The COVID-19 emergency has changed everyday life and we should take advantage of the lessons that we are learning from the experience, including building a healthcare system that is better able to protect the most fragile people in our society.

The COVID-19 outbreak has also seen an unprecedented collaboration between the public and private sectors that should be taken as a model for future synergies in the area of NCDs. The opportunity to engage older persons with NCDs to maintain cognitive and physical functioning and reduce solitude emerging from isolation should be also addressed. Now, more than ever, a holistic approach to NCD patients (that involves General Practitioners, Geriatric and Internal Medicine Physicians, Epidemiologists, Psychiatrists, and Behavioral Psychologists etc.) is mandatory, and rapid solutions to support this must be identified and implemented with urgency. We also recognize the pivotal role of Patient Advocacy Associations to define and implement supportive action that properly address patients’ needs. Partnership models like Project chAnGE can help achieve this goal, as it is based on collaboration between healthcare partners from various fields from private and public sectors. The project aims to identify challenges and solutions to healthy ageing in people with NCDs in Europe [38] and previous initiatives have emphasized the importance of Information and Communication Technologies and integrated care solutions in the management of NCDs for promoting healthy ageing [34, 39].
